# The critical impact of tumor size in predicting cancer special survival for T3aM0M0 renal cell carcinoma: A proposal of an alternative T3aN0M0 stage

**DOI:** 10.1002/cam4.3629

**Published:** 2020-12-06

**Authors:** Luping Li, Lei Shi, Junjie Zhang, Yingzhong Fan, Qi Li

**Affiliations:** ^1^ Department of Pediatric Surgery the First Affiliated Hospital of Zhengzhou University Zhengzhou University Zhengzhou China; ^2^ Department of Urology the First Affiliated Hospital of Zhengzhou University Zhengzhou University Zhengzhou China

**Keywords:** kidney carcinoma, prognostication, SEER, stage, TNM

## Abstract

**Objective:**

Based on the eighth TNM staging system, T3a renal cell carcinoma (RCC) is identified as an anatomical extrarenal invasion and does not consider the size of the tumor; however, it may not fully predict the prognosis of the patient. The objective of this study was to evaluate the prognostic value of tumor size effects on prognosis in T3a RCC and propose an alternative tumor stage system combined with T1‐2.

**Methods:**

Data relating to T1‐3aN0M0 RCC (n = 49586) were obtained from the Surveillance, Epidemiology, and End Results database (2004–2015). Survival analyses were conducted by Cox regression and Fine and Gray regression. Harrell's concordance index (c‐index) was used to assess the discriminatory ability of the prognostic factors.

**Results:**

A 1‐cm increase in T3a RCC resulted in an 8% increase in all‐cause mortality (hazard ratio [HR]: 1.08; 95% confidence interval [CI]: 1.06–1.10, *p* < 0.001) and 14% increase in the risk of RCC‐specific mortality (sub‐distribution HR [sHR]: 1.14; 95% CI: 1.11–1.16, *p* < 0.001). T3a tumor size stratified by the cutoff of 4 cm and 7 cm showed a better prediction of RCC‐special survival (c‐index: 0.644), compared with a cutoff just by 4 cm (c‐index: 0.571) or by 7 cm (c‐index: 0.602). Compared with T1b tumors, T3a RCC ≤4 cm showed no differences in terms of all‐cause mortality (HR: 0.93; 95% CI: 0.79–1.09; *p* = 0.37) and mortality caused by RCC (sHR: 0.91; 95% CI: 0.70–1.19; *p* = 0.50). Last, the alternative T‐staging system (T1a, a combination of T1b and T3a [≤4 cm], T2a, T2b, T3a [4–7 cm], and T3a [>7] cm) demonstrated good RCC‐special survival predictive accuracy (c‐index: 0.729), which was higher than that shown by the current eighth edition T‐staging system (c‐index: 0.720).

**Conclusion:**

Tumor size should be taken into consideration for T3aN0M0 RCC rather than based on anatomical features alone.

## Graphical abstract

Although the current TNM staging system demonstrated powerful prognostic discrimination, the use of the present system, some T3a RCCs with a small tumor size may give rise to overlapping prognoses with T1‐2 RCCs. Tumor size is an independent prognostic predictor and should be considered for improved survival stratification for locally advanced T3aN0M0 RCC rather than based on anatomical features alone

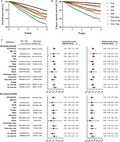

## 1 INTRODUCTION

The eighth edition of the American Joint Committee on Cancer (AJCC) Staging Manual was published in October 2016 and represents a compendium of all currently available information relating to the tumor‐node‐metastasis (TNM) staging of adult carcinomas in all clinically important anatomical sites.^1^ The T‐staging system for renal cell carcinoma (RCC) was defined according to tumor size and extrarenal invasion. The staging system for T3a RCC was revised in the eighth edition, as this type of tumor continues to be defined by anatomical extrarenal invasion, regardless of tumor size. Small and large RCCs can both exhibit extrarenal invasion and are thus classified together.^1^ A series of studies have reported that tumor size should be considered as an important parameter in the staging of T3a RCC and that a cutoff point of 7 cm may improve prognostic discrimination.^2^, ^3^, ^4^ Brookman–May et al^5^ further proposed that a 1‐cm increase in the size of a T3a tumor led to a 7% increase in mortality and that a cutoff point of 7 cm yielded the highest levels of predictive accuracy. However, it remains unknown as to whether the inclusion of tumor size would improve the prognostic discrimination of the current TNM staging system when classifying T3a tumors. To better evaluate the effect of different tumor sizes on the prognosis of T3a RCC patients, and to provide clinicians with a basis for judging the prognosis and offering the best treatment, we used the Surveillance, Epidemiology, and End Results [SEER] database to evaluate the survival outcome of this population.

## 2 PATIENTS AND METHODS

### 2.1 Patient cohort

We identified cases of T1‐3aN0M0 RCC (C64.9) from the SEER‐18 registry. The SEER database represents approximately 27.8% of the population in the USA, and the demographic characteristics of the SEER population are similar to the general population (https://seer.cancer.gov/). All patients were aged ≥18 years with RCC ≤15 cm and had undergone partial nephrectomy (PN) and radical nephrectomy (RN). In this study, we only included the three common histological types of RCC, namely clear‐cell RCC, papillary RCC, and chromophobe RCC. Patients were excluded if they had any other types of primary malignant tumors or had missing information concerning the cause of death. In addition, the follow‐up was <3 months and patients who died within 30 days were excluded.

### 2.2 Outcome and variables for analysis

The main endpoint events of interest were overall survival (OS) and cancer‐special survival (CSS). Death from other causes was considered a competing event, as it prevented mortality by RCC. The duration of survival was defined as the time from date of diagnosis to the date of death or last contact. We collated a number of demographic and tumor‐related variables in the study, including the age of diagnosis; year of diagnosis; sex; ethnicity; tumor size (cm); and histology of RCC (clear‐cell, papillary, or chromophobe); and nuclear grade (well‐differentiated [grade I], moderately differentiated [grade II], poorly differentiated [grade III], and undifferentiated [grade IV]).

### 2.3 Statistical analysis

Continuous variables and categorical variables are described as medians (interquartile range [IQR]) and frequencies (%), respectively. OS was compared using the Kaplan–Meier method for survival function along with the log‐rank test. Cumulative incidence was estimated using the competing risk time‐to‐event method and expressed as a percentage. The cumulative incidence function was then used to describe the incidence of RCC‐caused mortality. Univariable and multivariable Cox proportional hazards regression models were analyzed. Fine and Gray competing risks proportional hazard regression models were fitted to assess all‐cause mortality and RCC‐caused mortality. Harrell's concordance index (c‐index) was used to assess the discriminatory ability of the prognostic factors. The effect of tumor size on OS and CSS was assessed by flexible nonlinear Cox proportional hazard regression models using restricted cubic splines. We also evaluated the clinical scenario of an alternative and hypothetical T stage when a different cutoff for tumor size was considered as a parameter for T3a RCC. A nomogram was used to predict 5‐ and 10‐year CSS and a calibration method was used to assess the predictive accuracy of the nomogram. Prognostic markers included age, sex, histological type, grade, and tumor stage. Tumor size was not incorporated into the nomogram, because we considered this as a categorical variable. Discrimination was defined as the ability of our model to distinguish outcomes between different patients; in total, 1,000 bootstraps were used to validate our model. Calibration was defined previously as the ability of a model to yield unbiased estimates of the outcome, and the predictions of a well‐calibrated model should fall on a 45° diagonal line. All analyses were conducted using the R statistical package (v.3.5.2; R Foundation for Statistical Computing, Vienna, Austria; https://www.r‐project.org) and GraphPad Prism software version 6.0. All *P*‐values were two‐sided, and *p* < 0.05 was considered to indicate statistical significance.

## 3 RESULTS

### 3.1 Demographic characteristics, follow‐up, and outcomes

The study sample was a pooled cohort of 49,586 patients from the SEER database, of which, 6470 patients had T3a RCC. Table 1 presents the baseline characteristics of T3a RCC stratified by different tumor size groups, in general, larger tumors size have more chance of T3a stage, for tumor size more than 10 cm, there were 988 patients and it accounts for 15.3% of all T3a patients, compared with 188 (2.9%) tumor size less than 2 cm (Supplementary Table S1). Patients with T3a RCC had greater median age than those with T1 and T2 disease (63 years for T3a vs 59 years for T1 vs 58 years for T2). Further, the T3a group has more male patients than the T1 and T2 groups (69.2% for T3a vs 59.9% for T1 vs 64.3% for T2). Histologically, most T3a RCC cases were clear‐cell carcinoma (83.4%); whereas, only 76.4% and 71.2% T1 and T2 cases had clear‐cell carcinoma, respectively. Furthermore, more T3a RCC were of tumor grade G4 (9.4%) compared with 1.8% and 5.9% T1 and T2 cases, respectively (Table 1). In all, 43,53 (87.8%) T1‐3aN0 M0 patients were still alive, 2,516 (5.1%) patients died due to RCC, and 3,539 (7.1%) patients’ mortality was due to noncancer‐related causes during the median follow‐up period of 4.66 years, more T3a RCC patients were dead from the RCC (11.8%) compared with T1a (1.9%), T1b (5.2%), and T2a (9.9%), but less than T2b (13.1%) (Supplementary Table S2).

**TABLE 1:** Clinicopathological characteristics for 49586 RCC patients with tumor stages T1‐3aN0 M0.

	All T1‐3a	Tumor stage subgroups		
		T1	T2	T3a
Number of patients	(n = 49,586)	(n = 37,291)	(n = 5,825)	(n = 6,470)
Year of diagnosis				
2004–2007	11853 (23.9%)	9060 (24.3%)	1642 (28.2%)	1151 (17.8%)
2008–2015	37733 (76.1%)	28231 (75.7%)	4183 (71.8%)	5319 (82.2%)
Age at surgery (year)				
Mean/Median (IQR)	59/59 (50–68)	58.5/59 (50–67)	58.4/58 (50–67)	62.6/63 (55–71)
Age <50	11255 (22.7%)	8976 (24.1%)	1397 (24.0%)	882 (13.6%)
Age 50–64	21124 (42.6%)	15874 (42.6%)	2561 (44.0%)	2689 (41.6%)
Age 65–74	11675 (23.5%)	8600 (23.1%)	1261 (21.6%)	1814 (28.0%)
Age 75–84	4943 (10.0%)	3466 (9.3%)	527 (9.0%)	950 (14.7%)
Age ≥85	589 (1.2%)	375 (1.0%)	79 (1.4%)	135 (2.1%)
Sex, n (%)				
Female	19036 (38.4%)	14964 (40.1%)	2081 (35.7%)	1991 (30.8%)
Male	30550 (61.6%)	22327 (59.9%)	3744 (64.3%)	4479 (69.2%)
Ethnicity, n (%)				
White	40756 (82.2%)	30445 (81.6%)	4741 (81.4%)	5570 (86.1%)
Black	5110 (10.3%)	4039 (10.8%)	664 (11.4%)	407 (6.3%)
Other	3278 (6.6%)	2457 (6.6%)	376 (6.5%)	445 (6.9%)
Unknown	442 (0.9%)	350 (0.9%)	44 (0.8%)	48 (0.7%)
Histologic Type, n (%)				
Clear‐cell	38045 (76.7%)	28501 (76.4%)	4146 (71.2%)	5398 (83.4%)
Papillary	7459 (15.0%)	5962 (16.0%)	875 (15.0%)	622 (9.6%)
Chromophores	4082 (8.2%)	2828 (7.6%)	804 (13.8%)	450 (7.0%)
Grade, n (%)				
G1	5933 (12.0%)	5196 (13.9%)	419 (7.2%)	318 (4.9%)
G2	26323 (53.1%)	21009 (56.3%)	2657 (45.6%)	2657 (41.1%)
G3	12573 (25.4%)	8032 (21.5%)	1957 (33.6%)	2584 (39.9%)
G4	1615 (3.3%)	661 (1.8%)	344 (5.9%)	610 (9.4%)
Unknown	3142 (6.3%)	2393 (6.4%)	448 (7.7%)	301 (4.7%)
Tumor size (cm)				
Mean/Median (IQR)	4.7/4.0 (2.7–6.0)	3.6/3.5 (2.5–4.7)	9.4/9.0 (8.0–10.5)	6.9/6.5 (4.5–9.0)
Diameter ≤2	6900 (13.9%)	6712 (18.0%)	NA	188 (2.9%)
Diameter 2.1–3	9922 (20.0%)	9456 (25.4%)	NA	466 (7.2%)
Diameter 3.1–4	8801 (17.7%)	8134 (21.8%)	NA	667 (10.3%)
Diameter 4.1–5	6956 (14.0%)	6113 (16.4%)	NA	843 (13.0%)
Diameter 5.1–6	5065 (10.2%)	4237 (11.4%)	NA	828 (12.8%)
Diameter 6.1–7	3349 (6.8%)	2639 (7.1%)	NA	710 (11.0%)
Diameter 7.1–8	2841 (5.7%)	NA	2111 (36.2%)	730 (11.3%)
Diameter 8.1–9	1906 (3.8%)	NA	1336 (22.9%)	570 (8.8%)
Diameter 9.1–10	1292 (2.6%)	NA	812 (13.9%)	480 (7.4%)
Diameter >10	2554 (5.2%)	NA	1566 (26.9%)	988 (15.3%)
Surgery type				
Partial nephrectomy	18766 (37.8%)	17399 (46.7%)	389 (6.7%)	978 (15.1%)
Radical nephrectomy	30820 (62.2%)	19892 (53.3%)	5436 (93.3%)	5492 (84.9%)

Abbreviations: RCC, renal cell carcinoma; IQR, interquartile range.

Continuous variables and categorical variables are described as medians (interquartile range [IQR]) and frequencies (%), respectively.

### 3.2 The impact of tumor size on the prognosis for T3aN0M0 RCC

Supplementary Figure S1 showed the difference in the 5‐, 10‐years OS, and CSS of T1, T2, and T3a stages RCC between different tumor sizes groups. As the tumor size increases, the 5‐, 10‐years OS, and CSS in the same T stage are worse. Besides, T3a RCC compared with T1‐2 RCC with tumors of a similar diameter showed an inferior prognostication, but the small diameter of the T3a RCC has a better prognosis than the large‐diameter of T1‐2 RCC. The relationship between the log‐hazard ratio and tumor size was furtherly investigated (Supplementary Figure S2). Coefficients for HRs from Cox proportional hazard models of RCC‐specific mortality and all‐cause mortality were calculated using univariate and multivariate restricted cubic spline regressions. The effect of increasing tumor size on RCC‐caused mortality and all‐cause mortality was greatest for T3a RCC between 4~7 cm and smaller for tumors <4 cm and >7 cm, which suggested the tumor diameter does not conform to a linear relationship in predicting the OS and CSS of T3a RCC, therefore, it is necessary to analyze the diameter as a categorical variable. Combined with the current TNM system, we defined the 4 and 7 cm could be considered as a cutoff.

### 3.3 Survival analysis for tumor size as a predictive variable for patients with T3aN0M0 RCC

**FIGURE 1:**
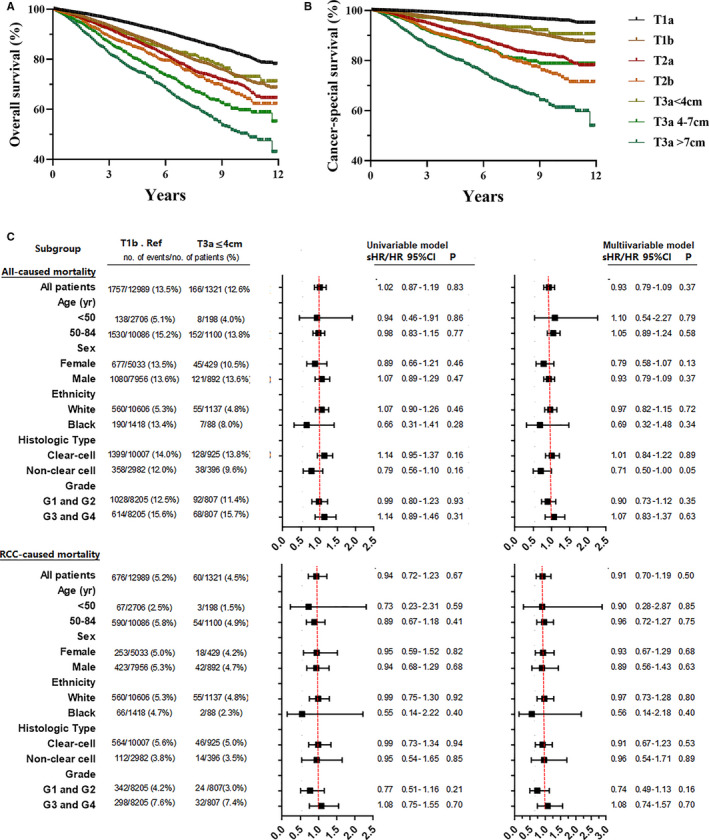
Kaplan–Meier analysis for overall survival (A) and cancer‐special survival (B) stratified according to tumor stage [T1a, T1b, T2, T3a (≤4 cm), T3a (4–7 cm) and T3a (>7 cm)]. Subgroup analysis of all‐cause mortality using the Cox proportional hazards regression model, and RCC‐caused mortality using the Fine and Gray competing risks proportional hazards regression model (C). The hazard ratio (HR) was derived from the Cox model, while the sub‐distribution HR (sHR) was derived from the Fine and Gray model (C). In the multivariable mordels, all other covariables were adjusted for each subgroup

For the entire T3a RCC cohort, a 1‐cm increase in T3a tumor size resulted in an 8% increase in all‐cause mortality (adjusted HR: 1.08; 95% CI: 1.06–1.10, *p* < 0.001) and 14% increase in mortality caused by RCC (adjusted sub‐distribution HR [sHR]: 1.14; 95% CI: 1.11–1.16, *p* < 0.001), respectively (Table 2) For cohort of T3a≤4 cm, the tumor size (per 1‐cm increase) did not increase the risk of mortality by both all‐cause (adjusted HR: 0.93, 95% CI: 0.73–1.18, *p* = 0.85) and RCC‐related (adjusted HR: 0.85, 95% CI: 0.56–1.28, *p* = 0.44). Besides, for cohort of T3a RCC >7 cm, a significant decrease was noted in the OS (adjusted HR: 1.08, 95% CI: 1.04–1.12, *p* < 0.001) and CSS (adjusted HR: 1.26, 95% CI: 1.05–1.14, *p* < 0.001) (Table 2). Figure 1A,B shows a comparable OS and CSS between patients with T3a RCC (≤4 cm) and T1b RCC. Compared with cohort of T1b RCC patients, T3a RCC (≤4 cm) had no significantly higher risk for all‐cause mortality (adjusted HR: 0.93; 95% CI: 0.7–1.09; *p* = 0.37) and mortality caused by RCC (adjusted sHR: 0.91; 95% CI: 0.70–1.19; *p* = 0.50) (Figure 1C).

**TABLE 2:** Tumor size considered as a predictor of outcomes in the cohort of T3a RCC patients and cohort of different tumor size groups (T3a≤4 cm, 4 cm<T3a≤7 cm, and T3a≥7 cm)^a^.

Different analysis cohorts	All‐cause mortality^b^			RCC‐caused mortality^c^			Competing events^c^		
	HR	95% CI	*p*	sHR	95% CI	*p*	sHR	95% CI	*p*
Univariate analysis results									
Entire T3a cohort	1.09	1.07‐1.11	<0.001	1.17	1.14‐1.19	<0.001	0.95	0.92‐0.98	<0.01
T3a≤4 cm cohort	1.03	0.84‐1.25	0.80	0.95	0.64‐1.42	0.80	1.22	0.92‐1.61	0.17
4 cm<T3a≤7 cm cohort	1.15	1.03‐1.28	0.02	1.29	1.12‐1.49	<0.001	0.96	0.83‐1.11	0.55
T3a≥7 cm cohort	1.08	1.04‐1.12	<0.001	1.11	1.06‐1.15	<0.001	0.96	0.89‐1.05	0.39
Multivariate analysis results^d^									
Entire T3a cohort	1.08	1.06‐1.10	<0.001	1.14	1.11‐1.16	<0.001	0.96	0.93‐0.99	0.02
T3a≤4 cm cohort	0.93	0.73‐1.18	0.54	0.85	0.56‐1.28	0.44	1.00	0.75‐1.34	0.99
4 cm<T3a≤7 cm cohort	1.09	0.98‐1.22	0.11	1.26	1.07‐1.47	<0.01	0.95	0.82‐1.11	0.51
T3a≥7 cm cohort	1.07	1.03‐1.11	<0.001	1.10	1.05‐1.14	<0.001	0.98	0.91‐1.06	0.64

Abbreviations: CI, confidence interval; HR, hazard ratio; RCC, renal cell carcinoma; sHR, Sub‐distribution hazard ratio.

^a^ Tumor size as a continuous predictive variable (per 1 cm) for survival functions based on a Cox model and Fine and Gray competing risks proportional hazards regression models, (345 patients with T3a RCC with unknown ethnicity and/or unknown grade were excluded from the multivariate analysis).

^b^ Cox proportional hazards regression model.

^c^ Fine and Gray competing risks proportional hazards regression model.

^d^ Tumor size adjusted for all other variables (year of diagnosis, age, sex, ethnicity, histology, and tumor nuclear grade) for multivariate analysis.

### 3.4 Derivation of a revised staging system for T1‐3aN0M0 RCC based on tumor size considered in T3aN0M0 and its value for prognostic discrimination

**FIGURE 2:**
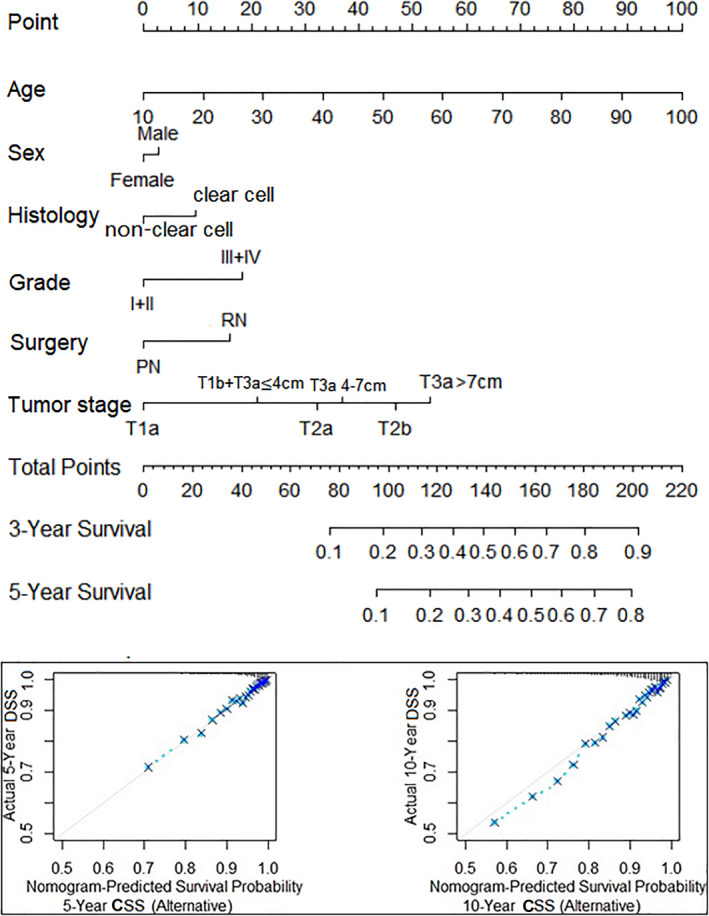
A nomogram for cancer‐specific survival (CSS) established using predictors and an alternative tumor staging system that combined T1b and T3a (≤4 cm), and a calibration plot of the nomogram predicting CSS at 5 and 10 years

Supplementary Table S2 showed the risk predators for mortality by RCC, we found age, sex, tumor grade, RCC histology, surgery, and alternative T stage were the independent predictors for RCC‐specific survival. Also, we found that the inclusion of tumor size into the regress model can increase the predictive power for OS and CSS prediction, if we did not include the tumor size as a predictor, the c‐index showed a slight decrease for the whole model (Table 3), Incorporating the cutoff of 4 cm and 7 cm for T3a RCC into the model for predicting CSS can obtain the greatest discriminatory ability (c‐index: 0.731). Based on these predictors, we developed a nomogram (Figure 2). The nomogram combined with the alternative T‐staging system (T1a, a combination of T1b and T3a [≤4 cm], T2a, T2b, T3a [4–7 cm], and T3a [>7 cm]) demonstrated good discrimination (c‐index: 0.799, compared 0.789 of the current T stage [T1a, T1b, T2a, T2b, and T3a] in the model, Table 3 and Figure 2).

**TABLE 3:** Discriminatory ability (Harrell's concordance index [c‐index]) of tumor stage in predicting survival in renal cell carcinoma.

	Univariate		Multivariate^a^	
	OS	CSS	OS	CSS
T3a renal cell carcinoma cohort				
T3a size as continue variables	0.570	0.621	0.695	0.727
T3a size as categorical variables				
T3a≤4 cm, T3a>4 cm	0.547	0.571	0.691	0.716
T3a≤4 cm, T3a= 4 to 7 cm, T3a>7 cm	0.583	0.644	0.698	0.731
T3a≤7 cm, T3a>7 cm	0.533	0.602	0.694	0.724
Without tumor size included in the multivariable model			0.686	0.707
T1‐3a renal cell carcinoma cohort				
The current T staging system				
T1a, T1b, T2a, T2b, T3a	0.614	0.720	0.724	0.789
Alternative T staging system−1				
T1a, T1b, T2a, T2b, T3a≤4 cm, T3a>4 cm	0.616	0.726	0.730	0.797
Alternative T staging system−2				
T1a, T1b, T2a, T2b, T3a≤4 cm, T3a= 4 to 7 cm, T3a>7 cm	0.616	0.728	0.730	0.798
Alternative T staging system−3				
T1a, T1b, T2a, T2b, T3a≤7 cm, T3a>7 cm	0.615	0.725	0.730	0.796
Alternative T staging system−4				
T1a, T1b+T3a≤4 cm, T2a, T2b, T3a= 4 to 7 cm, T3a>7 cm	0.616	0.729	0.730	0.799

Abbreviations: CSS, cancer special survival; OS, Overall survival.

^a^ adjusted for all other variables (year of diagnosis, age, sex, ethnicity, histology, and tumor grade) for multivariate cause‐special Cox regression analysis, and the c‐index was used to assess the discriminatory ability of the whole model.

## 4 DISCUSSION

This study led to several important findings. First, local extrarenal invasion (T3a) was linked to inferior prognostication for RCC patients with tumors of similar diameter (T1‐2). Second, in patients with T3aN0M0 RCC, tumor size was identified as an independent prognostic predictor. Our data suggest that it is reasonable to stratify small and large tumors in T3aN0M0 RCC in a manner that is not solely based on the extrarenal invasion. Third, the OS and cumulative incidence of RCC‐caused mortality in patients with T3aN0M0 (tumor size, ≤4 cm) RCC were equal to those of patients with T1bN0M0 RCC. Therefore, it is appropriate to merge T3aN0M0 (≤4 cm) and T1bN0M0 within the same stage classification; this merger did not reduce the predictive power of the prognostic model when tumor size was used as a staging parameter for T3aN0M0 RCC. These findings may have critical implications for the next revision of the TNM staging system and provide clinicians with important guidelines for prognostication and consulting for RCC patients.

An increasing number of asymptomatic small RCC tumors are being detected because of the widespread use of imaging (ultrasound and computed tomography). Moreover, the upstaging of small RCC (T1) to T3a is becoming more common.^6^, ^7^, ^8^, ^9^, ^10^, ^11^ Previous multicenter studies performed by Lam et al. and Brookman–May et al^3^, ^5^ showed that tumor size was an important factor for the prediction of outcome in patients with T3a RCC. These authors reported that T3a RCC tumors ≤7 cm showed no significant difference in terms of CSS when compared with T2. In the earlier studies, Cox proportional hazards regression models were used to analyze the endpoint of CSS. However, these authors did not consider the occurrence of competing events which precludes the observation of primary events of interest (RCC‐caused mortality) in real‐world clinical practice.^12^ In our study, T3a RCC tumors that were 4–7 cm in size were associated with a higher risk of mortality by RCC (data not shown); this was due to competing events before death that were related to RCC.

Patients with T1b and T3a RCC of ≤4 cm shared a similar probability of events related to RCC‐caused mortality. Besides, these patients were linked to similar risks of death that were related to competing events. Our results are consistent with those reported by Sugiyama et al.^13^ Furthermore, in the Sugiyama et al. study, the recurrence‐free survival for patients with T3a RCC (≤4 cm) was similar to that observed for patients with T1b RCC. Therefore, a comparable prognostic outcome was noted between stage T1b and T3a (≤4 cm). Hence, it is reasonable to combine both these T stages into a single alternative tumor stage classification. Furthermore, we observed a good prognostication accuracy when considering tumor size as a parameter for T3a RCC.

The current definition of T3a RCC is tumor grossly extends into the renal vein or its segmental (muscle‐containing) branches or tumor invades perirenal and/or renal sinus fat (peripelvic fat), but not beyond Gerota fascia. The three types of T3a infiltration (renal vein infiltration, perirenal fat, and renal sinus fat infiltration) may have their prognostic differences, and there are some pieces of evidence to prove this point.^14^, ^15^, ^16^ One of the most classic studies is from Shah's research,^14^ they found that the presence of multiple patterns of extrarenal extension (renal vein infiltration, perirenal fat, and renal sinus fat infiltration) is associated with a higher risk of disease progression and RCC‐related death after RN compared to isolated involvement of the renal vein infiltration, perirenal fat, and renal sinus fat infiltration. Because the information on this aspect in the SEER database is lacking, or only a small part of the information about fat infiltration around the kidney and renal sinus infiltration is recorded, and there is no vascular infiltration,^15^ so it cannot guarantee high‐quality regression analysis. In all, our conclusions can only be based on the results of the current eighth edition AJCC TNM RCC staging system.

It is not uncommon that the tumor upstaging from clinical T1 or T2 to T3a pathologically confirmed after PN.^17^ In the present study, the incidence of pathological upstaging from clinical T1 was 9.93%, consistent with that reported in recent studies (5%–14%).^8^, ^9^, ^10^, ^18^ The upstaging of pathological T stage after PN poses a dilemma for surgeons regarding the next surgical decision‐making (continually clinic observation following PN or an aggressive conversion to RN). The “Trifecta” concept for nephron‐sparing surgery includes the preservation of kidney function, oncological success, and a low incidence of urological complications. PN is widely used for the treatment of T1 RCCs; this has led to an overall increase in the incidence of T3a upstaging from T1 after PN. The current European Association of Urology [EAU], and the American Urological Association [AUA] guidelines do not recommend PN as a treatment option for T3a RCC, and there is no expression of any judgments for T3a management according to size. However, there are many reports in the literature that it is feasible to treat small T3a RCCs for PN,^19^, ^20^, ^21^, ^22^ which may have a certain reference value for future guideline changes. However, for patients with T3a RCC, performing PN surgery is a great challenge. It needs to be fully evaluated and informed of the possible risk of tumor control.

Consideration of tumor size as a parameter for T3a RCC may assist clinicians to select an optimum surgical approach for patients with smaller tumors that have been upstaged to T3a. Considering that conventional imaging techniques are limited in terms of detecting features relating to perirenal fat invasion, the situation of such a dilemma was not uncommon. A prior retrospective study conducted by Lee et al^19^ reported that for T3a RCC, patients who underwent PN versus RN showed no significant differences concerning DSS, or OS, and recurrence‐free survival. Furthermore, Jong et al.^21^ showed a consistent result that PN versus RN provided a comparable recurrence‐free survival in patients with small T3a tumors. All these studies suggested that it is necessary to stratify the T3a RCC based on tumor size rather than just considering the anatomic extrarenal extension.

T3a is a locally advanced stage of RCC, and there is a higher risk of recurrence and progression after surgery than RCCs of the same size without local extrarenal extension. The upstaging of T1 RCC to T3a is associated with adverse clinicopathological features. Indeed, several factors have been reported as preoperative risk factors for upstaging, including a higher R.E.N.A.L. score, advanced age, higher Fuhrman grade, tumor size, and male sex.^6^, ^8^, ^10^ A positive surgical margin is the main cause of local tumor recurrence and progression; nevertheless, this does not influence DSS, although there is an increased risk of recurrence.^23^ The incidence of a positive surgical margin is high after PN for T3a and ranges between 2.2% and 18.6% in the current literature.^6^, ^27^ A previous study reported that a positive margin was associated with a 3.08‐fold higher risk of recurrence than a negative margin when adjusted for surgical type.^8^ This indicated that radical tumor removal to maintain a negative margin was more important for the prevention of recurrence. Moreover, robot‐assisted PN has been reported to achieve optimal local cancer control in cases where there was a preoperative suspicion of T3a RCC.^24^

Finally, we proposed an alternative T stage system based on the tumor size of T3a with a cutoff of 4 cm and 7 cm, and combined T3a <4 cm with T1b, because two‐stage showed a nonsignificant survival difference, and we identified that the new alternative T stage system showed better prediction than the current eighth AJCC system for T stage. We further conducted a sensitivity analysis including the data of T3aNanyMany and found that stratification for T3a RCC with a cutoff of 4 cm and 7 cm still showed a good predictive accuracy (c‐index: 0.781, data not shown). Another study by our group showed that multiple patterns of perirenal fat invasion are associated with a poorer prognosis than isolated invasion, which was consistent with previous studies.^15^ Therefore, there is a need to further refine the reclassification for T3a RCC to increase the ability to predict prognosis and integrate tumor size and perirenal fat invasion types in future investigations.

Our study has some limitations. First, some of the data included in this study were obtained from the SEER database; hence, the retrospective nature of this investigation carries some inherent bias. In our study, we did not include cases with missing information concerning the cause of death, it may introduce a selection bias. Missing values in the data may be unavoidable in the retrospective study. Most patients in SEER database have records of overall survival outcome events but lack some specific causes of death, so the risk of cancer‐specific death may be biased. Although we have performed data selection, we try to include eligible cases in the study to avoid the emergence of selection bias as much as possible. Besides, we further conducted a sensitivity analysis on the data (data not shown) without deleting these cases and found that the results were not much different from the current research, and did not affect the conclusions. The probability of being N^+^ and M^+^ in T3a (also for <4 cm patients) might be higher than in T1‐2, but in our study, we exclude N^+^ and M^+^ from the study cohort, which also might have introduced a selection bias. To solve this question, we continued to conduct a rough analysis included the cohort of N^+^ and M^+^, and we found that the conclusion can still be verified (data not shown). Moreover, histopathological features lacked a centralized pathological review, time of recurrence, and treatment of recurrent disease. Despite these limitations, our findings are clinically important because they demonstrate that tumor size is a potential prognostic predictor for T3a RCC. Further studies are now needed to investigate additional modifications of the TNM staging system to improve prognostic discrimination.

## 5 CONCLUSIONS

Our analysis showed that the current TNM staging system demonstrated powerful prognostic discrimination. However, the use of the present system, some T3a RCCs with a small tumor size may give rise to overlapping prognoses with T1‐2 RCCs. For better clinical practice, we should consider sub‐staging the current T3a RCC stage by tumor size; this practice may improve surgical options. Furthermore, selected patients with small T3a RCCs may also receive treatment with PN instead of just an RN, as this practice may prolong OS. In short, tumor size is an independent prognostic predictor and should be considered for improved survival stratification for locally advanced T3aN0M0 RCC rather than based on anatomical features alone. Additional prospective studies should be conducted to overcome the limitations of our study and validate our findings.

## CONFLICT OF INTEREST

The authors declare to have no competing interest.

## AUTHORS CONTRIBUTIONS

Luping Li and Qi Li conceived the research and wrote the manuscript. Qi Li get the data. Lei Shi and Junjie Zhang analysis the data and prepare the figures and tables. All the authors were involved in approval of the final version.

## Supporting information

Supplementary Material

Supplementary Material

## Data Availability

The raw data of this study are derived from the SEER database (https://seer.cancer.gov/), which is a publicly available database. All detailed data included in the study are available upon request by contact with the corresponding author.
