# Supplementary material for: The critical impact of tumor size in predicting cancer special survival for T3aM0M0 renal cell carcinoma: A proposal of an alternative T3aN0M0 stage
Source: Cancer Med. 2020 Dec 6;10(2):605–14. doi: 10.1002/cam4.3629 (PMC7877365; doi:10.1002/cam4.3629)
Supplement: Supplementary file 1 — Supplementary Material [file CAM4-10-605-s001.docx]

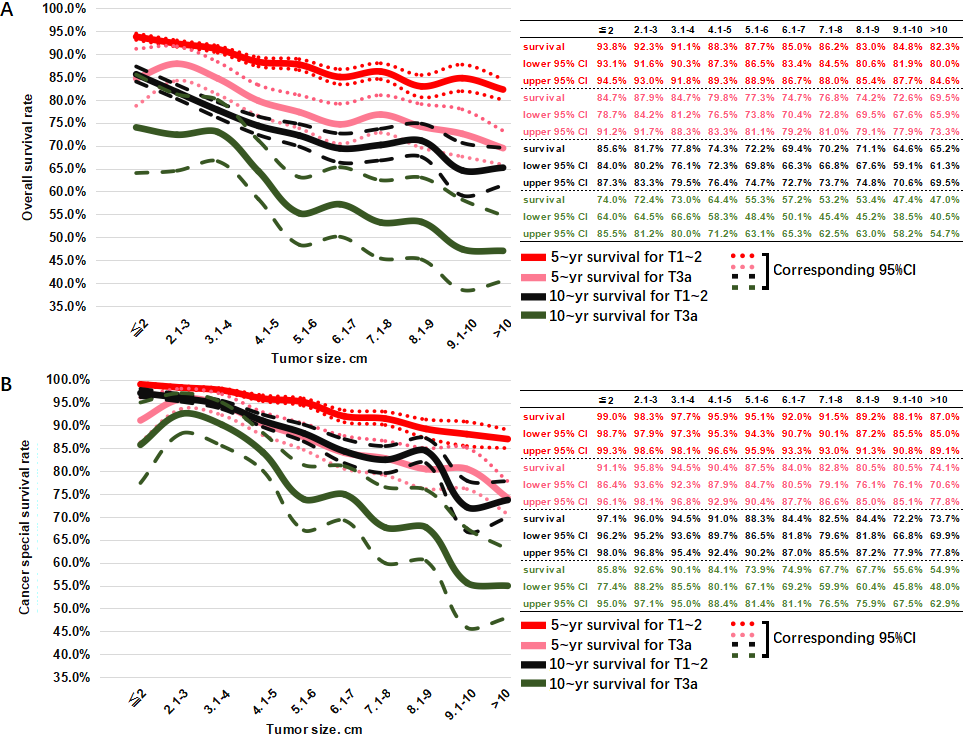


**Supplementary Figure 1.** *The survival rate, estimated by Kaplan–Meier analysis, between groups of different tumor sizes in patients with T1-3a renal cell carcinoma. (A) 5- and 10-year overall survival rate. (B) 5- and 10-year cancer-special survival rate with corresponding 95% confidence intervals (CIs). All P values were <0.01 when compared between T1-2 and T3a for 5- and 10-year survival.*


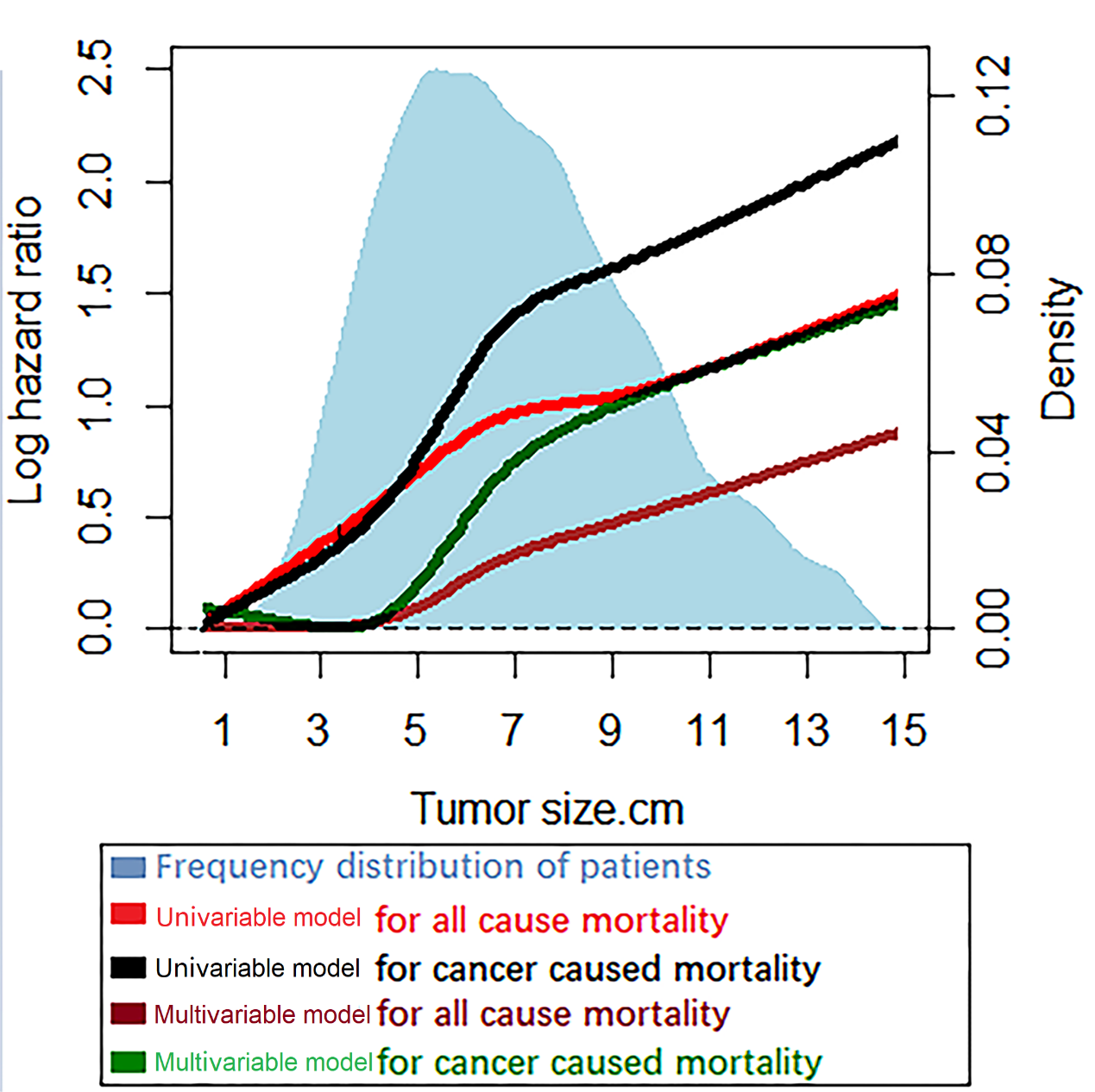


**Supplementary Figure 2.** Effect of tumor size as a continuous variable on the hazard ratio for all mortality and cancer-caused mortality in patients with T3a renal cell carcinoma. Results were derived from nuivariate and multivariate Cox proportional hazards models using restricted cubic splines with 5 knots at default percentile locations (5%, 27.5%, 50%, 72.5%, and 95%). The hazard ratio (HR) for tumor size in the multivariable model was adjusted for year at diagnosis, age at diagnosis, race, gender, histology type, and tumor grade.
