# Supplementary material for: The critical impact of tumor size in predicting cancer special survival for T3aM0M0 renal cell carcinoma: A proposal of an alternative T3aN0M0 stage
Source: Cancer Med. 2020 Dec 6;10(2):605–14. doi: 10.1002/cam4.3629 (PMC7877365; doi:10.1002/cam4.3629)
Supplement: Supplementary file 2 — Supplementary Material [file CAM4-10-605-s002.docx]

| **Supplementary Table 1. Clinicopathological characteristics for RCC patients with tumor stages T3aN0M0 stratified by tumor size.** | | | | | | | | | | | |
| --- | --- | --- | --- | --- | --- | --- | --- | --- | --- | --- | --- |
|  | **Overall** | **Tumor size stratification** | | | | | | | | | |
|  |  | **≤2cm** | **2.1–3cm** | **3.1–4cm** | **4.1–5cm** | **5.1–6cm** | **6.1–7cm** | **7.1–8cm** | **8.1–9cm** | **9.1–10cm** | **>10cm** |
| **Number of patients** | (N=6470) | (N=188) | (N=466) | (N=667) | (N=843) | (N=828) | (N=710) | (N=730) | (N=570) | (N=480) | (N=988) |
| ***Year of diagnosis*** |  |  |  |  |  |  |  |  |  |  |  |
| *2004–2007* | 1151 (17.8%) | 44 (23.4%) | 108 (23.2%) | 134 (20.1%) | 168 (19.9%) | 147 (17.8%) | 110 (15.5%) | 117 (16.0%) | 96 (16.8%) | 87 (18.1%) | 140 (14.2%) |
| *2008–2015* | 5319 (82.2%) | 144 (76.6%) | 358 (76.8%) | 533 (79.9%) | 675 (80.1%) | 681 (82.2%) | 600 (84.5%) | 613 (84.0%) | 474 (83.2%) | 393 (81.9%) | 848 (85.8%) |
| ***Age at surgery (year)*** |  |  |  |  |  |  |  |  |  |  |  |
| *Mean (SD)* | 62.6 (11.8) | 60.6 (11.5) | 62.1 (12.5) | 63.1 (11.9) | 63.9 (11.9) | 64.0 (11.8) | 63.2 (11.6) | 62.3 (11.5) | 62.6 (11.6) | 62.2 (11.2) | 60.6 (11.7) |
| *Age<50* | 882 (13.6%) | 29 (15.4%) | 82 (17.6%) | 87 (13.0%) | 94 (11.2%) | 96 (11.6%) | 86 (12.1%) | 90 (12.3%) | 79 (13.9%) | 66 (13.8%) | 173 (17.5%) |
| *Age 50–64* | 2689 (41.6%) | 83 (44.1%) | 168 (36.1%) | 258 (38.7%) | 338 (40.1%) | 326 (39.4%) | 300 (42.3%) | 337 (46.2%) | 237 (41.6%) | 201 (41.9%) | 441 (44.6%) |
| *Age 65–74* | 1814 (28.0%) | 50 (26.6%) | 137 (29.4%) | 201 (30.1%) | 242 (28.7%) | 243 (29.3%) | 203 (28.6%) | 181 (24.8%) | 159 (27.9%) | 143 (29.8%) | 255 (25.8%) |
| *Age 75–84* | 950 (14.7%) | 25 (13.3%) | 72 (15.5%) | 106 (15.9%) | 147 (17.4%) | 138 (16.7%) | 101 (14.2%) | 106 (14.5%) | 82 (14.4%) | 65 (13.5%) | 108 (10.9%) |
| *Age ≥85* | 135 (2.1%) | 1 (0.5%) | 7 (1.5%) | 15 (2.2%) | 22 (2.6%) | 25 (3.0%) | 20 (2.8%) | 16 (2.2%) | 13 (2.3%) | 5 (1.0%) | 11 (1.1%) |
| ***Sex, n (%)*** |  |  |  |  |  |  |  |  |  |  |  |
| *Female* | 1991 (30.8%) | 53 (28.2%) | 154 (33.0%) | 222 (33.3%) | 275 (32.6%) | 281 (33.9%) | 186 (26.2%) | 204 (27.9%) | 176 (30.9%) | 147 (30.6%) | 293 (29.7%) |
| *Male* | 4479 (69.2%) | 135 (71.8%) | 312 (67.0%) | 445 (66.7%) | 568 (67.4%) | 547 (66.1%) | 524 (73.8%) | 526 (72.1%) | 394 (69.1%) | 333 (69.4%) | 695 (70.3%) |
| ***Ethnicity, n (%)*** |  |  |  |  |  |  |  |  |  |  |  |
| *White* | 5570 (86.1%) | 169 (89.9%) | 400 (85.8%) | 568 (85.2%) | 730 (86.6%) | 714 (86.2%) | 625 (88.0%) | 617 (84.5%) | 500 (87.7%) | 424 (88.3%) | 823 (83.3%) |
| *Black* | 407 (6.3%) | 12 (6.4%) | 28 (6.0%) | 48 (7.2%) | 53 (6.3%) | 55 (6.6%) | 39 (5.5%) | 49 (6.7%) | 25 (4.4%) | 19 (4.0%) | 79 (8.0%) |
| *Other* | 445 (6.9%) | 6 (3.2%) | 31 (6.7%) | 43 (6.4%) | 55 (6.5%) | 52 (6.3%) | 43 (6.1%) | 59 (8.1%) | 43 (7.5%) | 31 (6.5%) | 82 (8.3%) |
| *Unknown* | 48 (0.7%) | 1 (0.5%) | 7 (1.5%) | 8 (1.2%) | 5 (0.6%) | 7 (0.8%) | 3 (0.4%) | 5 (0.7%) | 2 (0.4%) | 6 (1.2%) | 4 (0.4%) |
| ***Histologic Type, n (%)*** |  |  |  |  |  |  |  |  |  |  |  |
| *Clear-cell* | 5398 (83.4%) | 116 (61.7%) | 297 (63.7%) | 512 (76.8%) | 694 (82.3%) | 728 (87.9%) | 629 (88.6%) | 646 (88.5%) | 508 (89.1%) | 431 (89.8%) | 837 (84.7%) |
| *Papillary* | 622 (9.6%) | 54 (28.7%) | 116 (24.9%) | 100 (15.0%) | 85 (10.1%) | 63 (7.6%) | 48 (6.8%) | 43 (5.9%) | 32 (5.6%) | 25 (5.2%) | 56 (5.7%) |
| *Chromophores* | 450 (7.0%) | 18 (9.6%) | 53 (11.4%) | 55 (8.2%) | 64 (7.6%) | 37 (4.5%) | 33 (4.6%) | 41 (5.6%) | 30 (5.3%) | 24 (5.0%) | 95 (9.6%) |
| ***Grade, n (%)*** |  |  |  |  |  |  |  |  |  |  |  |
| *G1* | 318 (4.9%) | 19 (10.1%) | 44 (9.4%) | 63 (9.4%) | 44 (5.2%) | 31 (3.7%) | 31 (4.4%) | 28 (3.8%) | 22 (3.9%) | 13 (2.7%) | 23 (2.3%) |
| *G2* | 2657 (41.1%) | 108 (57.4%) | 245 (52.6%) | 328 (49.2%) | 404 (47.9%) | 367 (44.3%) | 284 (40.0%) | 278 (38.1%) | 218 (38.2%) | 142 (29.6%) | 283 (28.6%) |
| *G3* | 2584 (39.9%) | 43 (22.9%) | 124 (26.6%) | 227 (34.0%) | 308 (36.5%) | 328 (39.6%) | 310 (43.7%) | 333 (45.6%) | 234 (41.1%) | 214 (44.6%) | 463 (46.9%) |
| *G4* | 610 (9.4%) | 7 (3.7%) | 17 (3.6%) | 15 (2.2%) | 47 (5.6%) | 68 (8.2%) | 67 (9.4%) | 70 (9.6%) | 71 (12.5%) | 84 (17.5%) | 164 (16.6%) |
| *Unknown* | 301 (4.7%) | 11 (5.9%) | 36 (7.7%) | 34 (5.1%) | 40 (4.7%) | 34 (4.1%) | 18 (2.5%) | 21 (2.9%) | 25 (4.4%) | 27 (5.6%) | 55 (5.6%) |
| ***Tumor size (cm)*** |  |  |  |  |  |  |  |  |  |  |  |
| *Mean (SD)* | 6.87 (3.08) | 1.58 (0.387) | 2.64 (0.299) | 3.65 (0.289) | 4.66 (0.297) | 5.68 (0.298) | 6.66 (0.288) | 7.67 (0.299) | 8.68 (0.297) | 9.69 (0.301) | 12.1 (1.41) |
| ***Surgery type*** |  |  |  |  |  |  |  |  |  |  |  |
| *Partial nephrectomy* | 978 (15.1%) | 113 (60.1%) | 242 (51.9%) | 212 (31.8%) | 186 (22.1%) | 80 (9.7%) | 43 (6.1%) | 41 (5.6%) | 16 (2.8%) | 21 (4.4%) | 24 (2.4%) |
| *Radical nephrectomy* | 5492 (84.9%) | 75 (39.9%) | 224 (48.1%) | 455 (68.2%) | 657 (77.9%) | 748 (90.3%) | 667 (93.9%) | 689 (94.4%) | 554 (97.2%) | 459 (95.6%) | 964 (97.6%) |
| RCC=renal cell carcinoma; IQR=interquartile range. Continuous variables and categorical variables are described as mean (standard deviation [SD]) and frequencies (%), respectively. | | | | | | | | | | | |

| **Supplementary Table 2.** Follow-up time and status stratified by Tumor stages | | | | | | |
| --- | --- | --- | --- | --- | --- | --- |
|  | **T1-3a** | **T1a** | **T1b** | **T2a** | **T2b** | **T3a** |
| **Number of patients** | n=49586 | n=24302 | n=12989 | n=4259 | n=1566 | n=6470 |
| **Follow-up time (years)*** |  |  |  |  |  |  |
| Median | 4.66 | 4.70 | 4.92 | 5.17 | 5.08 | 3.83 |
| **Outcomes** |  |  |  |  |  |  |
| Alive and censored | 43531 (87.8%) | 22243 (91.5%) | 11232 (86.5%) | 3551 (83.4%) | 1276 (81.5%) | 5229 (80.8%) |
| Dead | 6055 (12.2%) | 2059 (8.5%) | 1757 (13.5%) | 708 (16.6%) | 290 (18.5%) | 1241 (19.2%) |
| Death due to RCC | 2516 (5.1%) | 450 (1.9%) | 676 (5.2%) | 423 (9.9%) | 205 (13.1%) | 762 (11.8%) |
| Dead due to non-RCC | 3539 (7.1%) | 1609 (6.6%) | 1081 (8.3%) | 285 (6.7%) | 85 (5.4%) | 479 (7.4%) |
| RCC=renal cell carcinoma  *The reverse Kaplan–Meier method was used to calculate the median follow-up time. | | | | | | |

| **Supplementary Table 3.** Hazard ratio (HR) of renal cell carcinoma related mortality | | | | | | | | |
| --- | --- | --- | --- | --- | --- | --- | --- | --- |
|  | **HR** | **95%CI lower** | **95%CI upper** | **P value** | **Adjusted HR** | **95%CI lower** | **95%CI upper** | **P value** |
| **Year of diagnosis** |  |  |  |  |  |  |  |  |
| 2004–2007 | 1 reference |  |  |  | 1 reference |  |  |  |
| 2008–2015 | 0.868 | 0.796 | 0.947 | < 0.001 | 0.836 | 0.765 | 0.913 | < 0.001 |
| **Age (per. 1 year)** | 1.046 | 1.043 | 1.05 | < 0.001 | 1.041 | 1.038 | 1.045 | < 0.001 |
| **Sex, n (%)** |  |  |  |  |  |  |  |  |
| Female | 1 reference |  |  |  | 1 reference |  |  |  |
| Male | 1.164 | 1.073 | 1.263 | < 0.001 | 1.092 | 1.005 | 1.188 | 0.038 |
| **Ethnicity, n (%)** |  |  |  |  |  |  |  |  |
| White | 1 reference |  |  |  |  |  |  |  |
| Black | 0.902 | 0.786 | 1.035 | 0.14 |  |  |  |  |
| Other | 1.033 | 0.883 | 1.207 | 0.688 |  |  |  |  |
| **Histologic Type, n (%)** |  |  |  |  |  |  |  |  |
| Clear-cell | 1 reference |  |  |  | 1 reference |  |  |  |
| Papillary | 0.809 | 0.721 | 0.907 | < 0.001 | 0.908 | 0.805 | 1.025 | 0.12 |
| Chromophores | 0.342 | 0.275 | 0.426 | < 0.001 | 0.338 | 0.271 | 0.423 | < 0.001 |
| **Grade, n (%)** |  |  |  |  |  |  |  |  |
| G1/G2 | 1 reference |  |  |  | 1 reference |  |  |  |
| G3/G4 | 2.904 | 2.678 | 3.149 | < 0.001 | 1.948 | 1.791 | 2.118 | < 0.001 |
| **Surgery type** |  |  |  |  |  |  |  |  |
| Partial nephrectomy | 1 reference |  |  |  | 1 reference |  |  |  |
| Radical nephrectomy | 4.097 | 3.619 | 4.638 | < 0.001 | 1.771 | 1.548 | 2.026 | < 0.001 |
| **T stage** |  |  |  |  |  |  |  |  |
| T1a | 1 reference |  |  |  | 1 reference |  |  |  |
| T1b+ T3a< 4cm | 2.855 | 2.539 | 3.211 | < 0.001 | 2.091 | 1.851 | 2.363 | < 0.001 |
| T2a | 5.356 | 4.69 | 6.116 | < 0.001 | 3.699 | 3.213 | 4.258 | < 0.001 |
| T2b | 7.288 | 6.178 | 8.597 | < 0.001 | 5.436 | 4.571 | 6.464 | < 0.001 |
| T3a 4-7cm | 6.964 | 5.951 | 8.148 | < 0.001 | 3.845 | 3.261 | 4.535 | < 0.001 |
| T3a>7cm | 12.781 | 11.221 | 14.558 | < 0.001 | 6.685 | 5.791 | 7.718 | < 0.001 |
| HR, Hazard ratio; CI, Confidence interval | | | | | | | | |
